# Oxidative DNA Damage-induced PARP-1-mediated Autophagic Flux Disruption Contributes to Bupivacaine-induced Neurotoxicity During Pregnancy

**DOI:** 10.2174/1570159X21666230404102122

**Published:** 2023-08-15

**Authors:** Jiaming Luo, Lei Zeng, Ji Li, Shiyuan Xu, Wei Zhao

**Affiliations:** 1 Department of Anesthesiology, Zhujiang Hospital, Southern Medical University, Guangzhou City, Guangdong Province, China;; 2 Division of Laboratory Science, Affiliated Cancer Hospital & Institute of Guangzhou Medical University, Guangzhou, China

**Keywords:** Autophagy flux, bupivacaine, neurotoxicity, PARP-1, pregnancy, neuronal DNA damage

## Abstract

**Objective:**

Severe neurologic complications after spinal anesthesia are rare but highly distressing, especially in pregnant women. Bupivacaine is widely used in spinal anesthesia, but its neurotoxic effects have gained attention.

**Methods:**

Furthermore, the etiology of bupivacaine-mediated neurotoxicity in obstetric patients remains unclear. Female C57BL/6 mice were intrathecally injected with 0.75% bupivacaine on the 18th day of pregnancy. We used immunohistochemistry to examine DNA damage after bupivacaine treatment in pregnant mice and measured γ-H2AX (Ser139) and 8-OHdG in the spinal cord. A PARP-1 inhibitor (PJ34) and autophagy inhibitor (3-MA) were administered with bupivacaine in pregnant mice. *Parp-1*^flox/flox^ mice were crossed with Nes-Cre transgenic mice to obtain neuronal conditional knockdown mice. Then, LC3B and P62 staining were performed to evaluate autophagic flux in the spinal cords of pregnant wild-type (WT) and *Parp-1*^-/-^ mice. We performed transmission electron microscopy (TEM) to evaluate autophagosomes.

**Results:**

The present study showed that oxidative stress-mediated DNA damage and neuronal injury were increased after bupivacaine treatment in the spinal cords of pregnant mice. Moreover, PARP-1 was significantly activated, and autophagic flux was disrupted. Further studies revealed that PARP-1 knockdown and autophagy inhibitors could alleviate bupivacaine-mediated neurotoxicity in pregnant mice.

**Conclusion:**

Bupivacaine may cause neuronal DNA damage and PARP-1 activation in pregnant mice. PARP-1 further obstructed autophagic flux and ultimately led to neurotoxicity.

## 1 INTRODUCTION

With trends in cesarean delivery and the popularization of painless delivery technology, more women receive spinal anesthesia. Local anesthetics are often used for spinal anesthesia in maternity wards. However, sensory and motor dysfunction have often been reported to be neurotoxic complications of local anesthetics due to their neurotoxic effects. Severe neurologic complications after spinal anesthesia are rare but highly distressing, especially in pregnant women [1]. Patients mainly exhibit bilateral pain in the buttocks and thighs [2]. This painful condition that occurs 24 hours postoperatively is termed transient neurological symptoms (TNS) [3]. The reported incidence of TNS after spinal anesthesia in the obstetric population ranges from 0 to 8.8% [2]. However, the etiology of TNS in obstetric patients is still unclear.

The increased neural sensitivity to local anesthetics contributes to a significant increase in peripheral neurological complications in pregnant women following spinal anesthesia [4]. Previous studies have indicated that oxidative stress is the critical factor in neurotoxic injury caused by the local anesthetic bupivacaine [5]. Other studies have shown that elevated oxidative stress levels during pregnancy are associated with pregnancy-related complications such as gestational diabetes mellitus, preeclampsia, and preterm birth [6]. Whether high maternal oxidative stress during pregnancy is the key to exacerbating the neurologic complications of local anesthetic has not been reported.

Oxidative stress is a key point in DNA damage. Oxidative DNA damage is an important threat to the genomic stability of mature neurons [7]. Our previous study showed that bupivacaine-mediated oxidative stress could lead to neuronal DNA damage and activate the base excision repair pathway and the essential enzyme poly (ADP-ribose) polymerase (PARP)-1 [8]. Usually, PARP-1 activity is low but is increased to catalyze the poly-ADP ribosylation of the DNA repair receptor proteins in response to DNA damage [9]. The local anesthetic bupivacaine can increase the production of autophagosomes and disrupt the clearance of autophagosomes, thus blocking the autophagic flow. Recent studies have suggested that PARP-1 is the primary upstream regulator of autophagy [10]. During oxidative stress and DNA damage, PARP-1 determines cell fate by regulating the dynamic balance between autophagy and necrosis [11]. It is unclear whether local anesthetics induce peripheral neurologic complications during pregnancy associated with PARP-1 regulation of autophagic flow.

Herein, we examined the effect of the local anesthetic bupivacaine on neurotoxic injury in a pregnant mouse model. Spinal cord neuronal injury in pregnant mice was measured by simulating spinal anesthesia. Thus, this study pursued two research objectives: 1) to explore whether oxidative stress, PARP-1, and autophagy are involved in neurotoxic damage caused by bupivacaine in pregnancy and 2) to investigate whether oxidative stress/PARP-1-mediated autophagic flux disruption is the mechanism of bupivacaine neurotoxicity during pregnancy.

## 2 MATERIALS AND METHODS

### 2.1 Chemicals and Reagents

PJ34 HCl and 3-methyladenine (3-MA) were acquired from Selleck Chem USA. Bupivacaine hydrochloride was purchased from Sigma-Aldrich (St. Louis, Missouri, USA). 2`, 7`-dichlorofluorescein diacetate (DCFH-DA) was acquired from Abcam (Cambridge, Massachusetts, UK). Antibodies against PARP-1, cleaved- PARP-1, P62, and cytochrome c were purchased from Cell Signaling Technology (Boston, MA, USA). An antibody against NeuN and LC3B was purchased from Merck Millipore (Darmstadt, Germany). The antibody against 8-OHdG was obtained from A&D Technology. Antibody against γ-H2AX (Ser139) was purchased from Affinity Biosciences. The secondary antibodies used were 1:500 anti-rabbit Alexa Fluor 488 and anti-mouse Alexa Fluor 594 from Thermos Fischer Scientific.

### 2.2 Animal Experiment

Adult female C57BL/6N mice weighing 25-30 g were used for this study. Mice were supplied by the Center of Animals of Southern Medical University, Guangzhou. The ethics committee approved all the procedures of Zhujiang Hospital of Southern Medical University of Medical Sciences (LAEC-2020-071), which followed the ethical standards guidelines for the care and use of laboratory animals for animal research. All animals were bred and maintained under standard husbandry conditions (22 ± 2°C, humidity 55-70%, and 12 h light/dark cycle). Mice were raised in cages with a female/male ratio of 2:1. On day 18 of pregnancy, pregnant mice were randomly allocated into groups for experiments.

### 2.3 Drug Delivery

The intrathecal injection was performed to deliver the drug or vehicle in this experiment with reference to a previous study [12]. To perform the intrathecal (*i.t.*) injections, the mice were placed in a prone position. Then, the midpoint between the tips of the iliac crest was located. A 30 G needle attached to a microsyringe was inserted between the L4 and L5 vertebrae in lightly restrained, unanesthetized mice for intrathecal drug injection. The reflexive tail flick indicated a successful puncture. The volumes of all injections were 5 µL of vehicle. Drugs were used at the following concentrations: 0.75% bupivacaine was used based on our previous experiences [5]. PJ-34 (a specific PARP-1 inhibitor) at 10 mg kg^-1^ was administered according to a previous study [13]. 3-MA (autophagy inhibitor) at 10 mg kg^-1^ was used based on previous experiences [14]. Drugs were dissolved in sterile normal saline and warmed with a 50°C water bath. One dose of PJ-34 or 3-MA was given intraperitoneally 60 min before the intrathecal injection of bupivacaine.

### 2.4 Generation of Neuronal Conditional Knockdown (*Parp-1^-/-^,* CKO) Mice

#### 2.4.1 Parp-1^-/-^

CKO mice were acquired from Cyagen Biosciences (Guangzhou, China, CKOAI191017MG1). Briefly, the gRNA to the mouse Parp-1 gene [15] (NCBI-ID: 11545), the donor vector containing the loxP sites, and Cas9 mRNA were coinjected into fertilized mouse eggs to generate targeted CKO offspring. F0 founder animals were identified by PCR, followed by sequence analysis, and bred to wild-type mice to test germline transmission and F1 animal generation. Then, *Parp-1*^flox/flox^ mice were crossed with Nes-Cre transgenic mice to obtain neuronal CKO mice.

### 2.5 Mechanical Thresholds and Thermal Withdrawal Latency

All mice were examined for mechanical thresholds and thermal withdrawal latency as described [16] before and after 0.75% bupivacaine injection. All behavioral observers were blinded to the experimental grouping. Mechanical thresholds were measured by the paw withdrawal threshold (PWT) with a set of Von Frey filaments (0.04-0.2 g; Ugo Basile, Gemonio, Italy). The filament is applied to the plantar surface of the left hind paw at a vertical angle for up to 3 s from the bottom. The 50% probability of the paw withdrawal threshold (MWT) was calculated using the up-down method.

The thermal withdrawal latency was measured by the paw withdrawal latency to radiant heat stimuli. Before the test, the mice were placed in elevated chambers on a Plexiglas floor and allowed to habituate for 30 min. The radiant heat source (plantar test, 37370; Ugo Bazile, SRL, Gemonian, Italy) was applied to the center of the plantar surface of the left hind paw with at least a 3-minute interval. The average withdrawal latency of these trials was deemed the response latency.

### 2.6 TdT-mediated dUTP Nick-end Labeling (TUNEL)

TUNEL was used to evaluate fragmented DNA and apoptosis. An *in-situ* cell death detection kit (Roche Molecular Biochemical, Germany) was used to measure DNA damage and apoptotic cells according to the kit’s instructions. The spinal cord tissues were fixed with 4% paraformaldehyde for 20 min and washed three times for 10 min per wash with PBS (pH 7.4). Then, the sections were permeabilized with 0.1% sodium citrate solution containing 0.1% Triton X-100. After 2 min in an ice bath, sections were incubated at 37^o^C for 1 h with the new reaction solution. After tissues were washed with PBS for 30 minutes, sections were incubated for 10 minutes in the dark for 4, 6-diamino-2-phenylindole (DAPI) staining. Immunostaining was visualized with a Nikon Eclipse Ti2 fluorescence microscope. DAPI nuclear staining was used to determine each area's total number of cells. The co-localization of both the TUNEL signal and DAPI identified TUNEL-positive cells. Then, the selected images were converted into 8-bit grayscale images. The integral optic density (IOD) of selected images was measured and calculated in Image-Pro Plus v6.0 software (Media Cybernetics Inc. Bethesda, MD, USA) [17].

### 2.7 Reactive Oxygen Species (ROS) Assay

ROS production from spinal cord samples was tested using the DCFH-DA dye from Abcam (Cambridge, Massachusetts, UK). Frozen sections (10 μm) of the spinal cord were washed with cleaning buffer for 5-10 min and then incubated with DCFH-DA at 37°C for 30 min. After DAPI staining, the ROS levels in the spinal dorsal horn were measured *via* fluorescence microscopy (Axio Vert A1, Carl Zeiss, Jena, Germany). The fluorescence intensity of the images was quantified using Image-Pro Plus v6.0 software (Media Cybernetics Inc. Bethesda, MD, USA).

### 2.8 Oxidative Stress Assay

The activities of superoxide dismutase (SOD) and the level of malondialdehyde (MDA) were used to evaluate oxidative stress. The MDA concentration and SOD activity in the spinal cord tissue were measured by an MDA kit (Nanjing Jiancheng Bioengineering Institute, China) and a SOD assay kit (Beyotime, Shanghai, China) as described previously [5]. Chemiluminescent absorbance was assessed with a microplate reader (Bio-Rad, USA). The tissue samples (10 mg spinal cord tissue + 100 volume SOD assay buffer) were prepared using SOD assay buffer, followed by making a master reaction mix. The reaction mixture (final volume 200 μL) consisted of 20 μL of the sample, 30 μL of WST-8 solution, and 20 μL of reaction start-up reagent. Then, the samples were incubated at 37°C for 30 min, and the activity was determined by measuring the absorbance change at 450 nm. The same samples (10 mg spinal cord tissue + 10 volume MDA assay buffer) were also prepared using MDA assay buffer. Following centrifugation, the absorbance of the supernatant was measured at 532 nm. SOD activity and the MDA concentration per microgram of protein for each sample were calculated. Inputs for samples from each trial were normalized by protein mass determined from BCA assays.

### 2.9 Histopathological Examination (H/E Staining)

Hematoxylin-eosin (H/E) staining was performed as previously described [5]. Briefly, spinal cord paraffin sections were baked at 60^o^C for 2 h and dewaxed and hydrated. Then, the slices were placed into distilled water and hematoxylin aqueous solution for staining for 3 min. Next, slices were rinsed with running water and stained with eosin-lasting solution (G1100; Solar bio) for 3 min. Finally, the slices were dehydrated, transparentized, and sealed. The sections were stained with H/E, and then images were observed and captured with a light microscope (Leica DM2500, Wetzlar, Germany).

### 2.10 Immunohistochemistry

Immunohistochemistry was performed according to the manufacturer’s protocol. Briefly, the tissues were fixed with 4% polyoxymethylene, embedded in paraffin, and sectioned at 5 µm thickness. Then, the sections were dewaxed in xylene (Sigma) and rehydrated in graded ethanol. Endogenous peroxidase was blocked by incubation in H_2_O_2_ (3%) for 10 min. Then, sections were incubated with anti-phosphorylation of γ-H2AX (Ser139) and 8-OHdG at 4°C overnight, followed by incubation with the secondary antibodies for 1 h at 25°C. The images were observed and captured with a light microscope (Leica DM2500, Wetzlar, Germany). The γ-H2AX (Ser139) and 8-OHdG expression levels were represented by IOD. The selected images were converted into 8-bit grayscale images. Then, the IODs of each image was measured and calculated in Image-Pro Plus v6.0 software (Media Cybernetics Inc. Bethesda, MD, USA).

### 2.11 Immunofluorescence

Immunostaining was performed on frozen tissue sections on glass coverslips and fixed with 4% paraformaldehyde for 30 min. Then, the sections were permeabilized with 0.5% Triton X-100 for 15 min. After blocking with 5% normal goat serum in PBS buffer for 1 h at 25°C, the slides were washed with PBS three times for 10 min per wash. Then, the sections were incubated with primary antibodies overnight at 4°C. The secondary antibody was added the next day. DAPI (1:10,000, Sigma-Aldrich) was used to stain nuclei. The images were observed and captured with a fluorescence microscope (Nikon Instruments, Japan). Finally, the IODs of each image was measured and calculated in Image-Pro Plus v6.0 software (Media Cybernetics Inc., Bethesda, MD, USA).

### 2.12 Western Blotting

Western blotting was performed as described in a previous study. Briefly, spinal cord tissue was lysed by sonication in precooled lysis buffer. Protein concentration was determined by the BCA method for Western blotting. Equal amounts of total protein (60 μg) were separated by 10% SDS-PAGE and transferred onto a PVDF membrane (Immobilon-P, Millipore, and Bedford, MA, USA). After blocking with 5% nonfat dry milk at room temperature for 1 hour, the blots were incubated overnight at 4^o^C with primary antibodies against PARP-1, cleaved-PARP-1 (rabbit, 1:1000), and α-tubulin (rabbit, 1:2000). Then, all blots were developed with HRP-conjugated secondary antibodies and ECL solution (GE Healthcare, Uppsala, Sweden). The blots were visualized in ECL solution for 1 min, and images were captured on a Luminescent Image Analyzer (ChemiDoc Imaging Systems, Bio-Rad Laboratories, USA). The intensity of the selected bands was captured and quantified by a densitometric method with Image J software 1.48a (http://imagej.nih.gov).

### 2.13 Transmission Electron Microscopy (TEM)

TEM was used to measure the autophagosomes as described previously [18]. *Parp-1 wild-type* (*wt)* and pregnant knockout mice were used to determine the differences in autophagy induction after bupivacaine injection. The spine cord from *wt.* and *Parp-1* CKO mice were extracted and washed with PBS buffer (pH = 7.4) and then prefixed for 30 min in a fixation solution (0.1 M cacodylate buffer pH 7.4 and osmium tetraoxide) for 60 min at 4°C. Then, the tissues were washed with Milli-Q water, and the samples were stained with uranyl acetate. Ultrathin sections were cut with ultramicrotome using a diamond knife (Reichert Ultra cut S). The sections were observed and captured with a TEM Zeiss 902 with 80 kV voltage acceleration (CIC-UGR).

### 2.14 Statistical Analysis

No statistical methods were applied in this study to predetermine the sample sizes. However, our sample sizes are similar to those reported in previous studies. All graphs and statistical calculations were generated using Graph Pad Prism 8.0 (Graph Pad Software Inc., La Jolla, CA). Analyses were performed blinded to the genotype and experimental group. *P*-values are depicted in the figure legends with corresponding symbols on the graphical data. We first calculated the Gaussian distribution of the data using the Shapiro-Wilk test. An unpaired t-test (Gaussian distribution) was used when two groups were compared. When continuous variables across multiple groups were compared for several parameters, we used a one- or two-way ANOVA followed by Tukey’s (Gaussian distribution) post-hoc tests. *P* < 0.05 was considered significant.

## 3 RESULTS

### 3.1 Bupivacaine Could Induce Spinal Cord Neuronal Injury and Behavioral Changes in Pregnant Mice

**Fig. (1):**
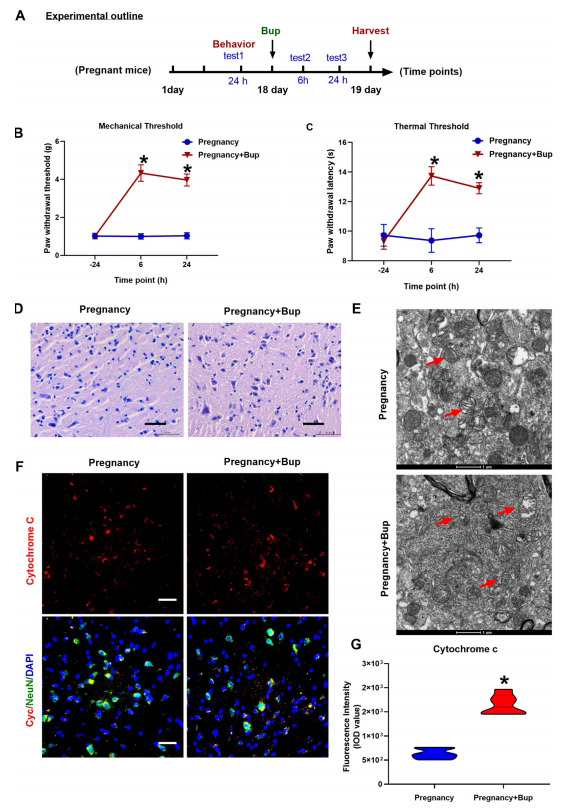
Bupivacaine could induce spinal cord neuronal injury and behavioral changes in pregnant mice. (**A**) Outline of the experimental design. C57BL/6 female mice were intrathecally administered 5 µL of 0.75% bupivacaine on the 18th day of pregnancy. At twenty-four hours before and six and twenty-four hours after bupivacaine injection, the behavioral tests for mechanical (**B**, *p < 0.001) and thermal thresholds (**C**, *p < 0.001) were performed. (**D**) H/E staining of the spinal cord. Spinal cords were stained with H/E and imaged by light microscopy. Scale bar: 50 µm. (**E**, **F**, *p = 0 .0013) Immunostaining of cytochrome c. The spinal cord was probed with anti-cytochrome c (red fluorescence). NeuN (Green) was used to label mature neurons, whereas the cell nuclei were stained with DAPI (blue). Scale bar, 50 µm. (**G**) Representative TEM images of the spinal cords of pregnant mice treated with or without bupivacaine. The red arrows indicate mitochondria. Scale bar: 1 µm. The results are presented as the mean ± SD (n = 6); *p compared with the pregnancy group.

C57BL/6 female mice were intrathecally injected with 0.75% bupivacaine on the 18^th^ day of pregnancy. Behavioral tests for mechanical and thermal thresholds were performed twenty-four hours before and six hours after the bupivacaine injection. The mechanical pain paw withdrawal threshold (PWMT) and reflexology photothermal stimulation of mouse latency (PWTL) values were both significantly increased compared to those in the pregnancy group (Figs. **1A**-**C**; *p < 0.001). The behavioral test results showed that a single intrathecal injection of bupivacaine elicited behavioral changes in pregnant mice. The H/E staining (Fig. **1D**) results showed significant atrophy, and the tissues surrounding the lesion area became looser in the spinal cords in the bupivacaine group. Cytochrome c and NeuN double immunofluorescence staining were used to detect neuronal injury in the spinal cords of the mice. As shown in Figs. (**1E** and **F**), bupivacaine markedly increased the production of cytochrome c in the spinal cord neurons of pregnant mice (fold change 2.67, *p = 0.0013). TEM was used to observe ultrastructural changes in mitochondria. As shown in Fig. (**1G**), more overt mitochondrial fragmentation with structural fragmentation was observed in the bupivacaine injection group than in the control group.

### 3.2 Oxidative Stress-mediated DNA Damage was Increased in Pregnant Mice after Bupivacaine Treatment

**Fig. (2):**
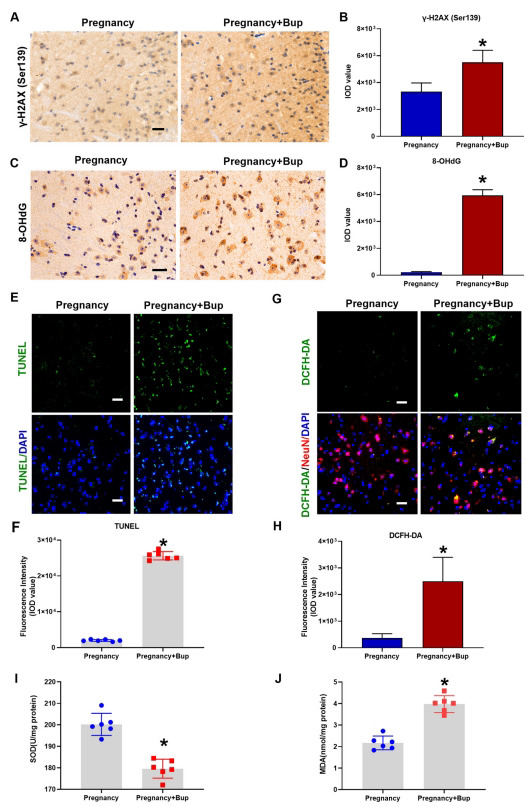
Oxidative stress-mediated DNA damage was increased in the spinal cord of pregnant mice after bupivacaine treatment. To assess DNA damage after bupivacaine treatment in the spinal cords of pregnant mice, we used immunohistochemistry to examine the phosphorylation of γ-H2AX (Ser139) (**A**, **B**, *p = 0.0007) and 8-OHdG (**C**, **D**, *p < 0.0001). (**E**, **F**, *p < 0.0001) TUNEL staining was performed to determine cell apoptosis and DNA damage in spinal cord tissues. The cell nuclei were stained with DAPI (blue). Scale bar, 50 µm. (**G**, *p = 0.0002) Immunostaining of ROS. DCFH-DA is a probe to measure ROS by fluorescence microscopy and demonstrated ROS production after bupivacaine treatment in the spinal cords of pregnant mice. The spinal cord was probed with DCFH-DA (green fluorescence). NeuN (red) was used to label mature neurons, and the cell nuclei were stained with DAPI (blue). Scale bar, 50 µm. (**H**) Quantitative analysis of the fluorescence intensity. (**I**, **J**) Quantitative analysis of MDA (*p = 0.0146) and SOD (*p = 0.0187) levels in the spinal cord tissues of each group. The results are presented as the mean ± SD (n = 6); *p compared with the pregnancy group.

Accumulating evidence has shown the critical role of DNA damage in neuronal function [19]. Our previous study indicated that bupivacaine could mediate neuronal DNA damage and apoptosis [20]. To assess DNA damage after bupivacaine treatment in the spinal cords of pregnant mice, we used immunohistochemistry to examine the phosphorylation of γ-H2AX (Ser139) and 8-OHdG. As shown in Figs. (**2A** and **2B**) (fold change 1.66, *p = 0.0007), bupivacaine increased the phosphorylation of γ-H2AX (Ser139) in the spinal cords of pregnant mice. Furthermore, the oxidative DNA damage marker 8-OHdG was also highly expressed (Figs. **2C** and **2D**; fold change 26, *p < 0.0001). Moreover, the TUNEL assay revealed that the percentage of apoptotic cells and DNA damage (Figs. **2E** and **2F**; fold change 13.1, *p < 0.0001) was significantly increased in the spinal cord tissue of the pregnancy and bupivacaine groups. ROS are the main products of oxidative stress [21]. During pregnancy, the production of ROS plays a vital role in triggering DNA damage. We used DCFH-DA as a probe and fluorescence microscopy to measure ROS production after bupivacaine treatment during pregnancy. As shown in Figs. (**2G** and **2H**) (fold change 6.82, *p = 0.0002), spinal cord tissue slices in the pregnancy and bupivacaine (Preg + Bup) group displayed increased ROS production in spinal neurons. SOD and MDA levels were used to evaluate oxidative stress in the spinal cord tissue. As shown in Figs. (**2I** and **J**) (fold change 1.76, *p = 0.0146) and J (fold change 0.89, *p = 0.0187), oxidative stress was increased in the spinal cord tissue in the pregnancy and bupivacaine (Preg + Bup) group. These data showed that bupivacaine increased oxidative DNA damage in the spinal cords of pregnant mice.

### 3.3 PARP-1 was Significantly Activated in Pregnant Mice by Treatment with Bupivacaine, and PARP-1 Inhibitors could Substantially Alleviate Neurotoxicity

**Fig. (3):**
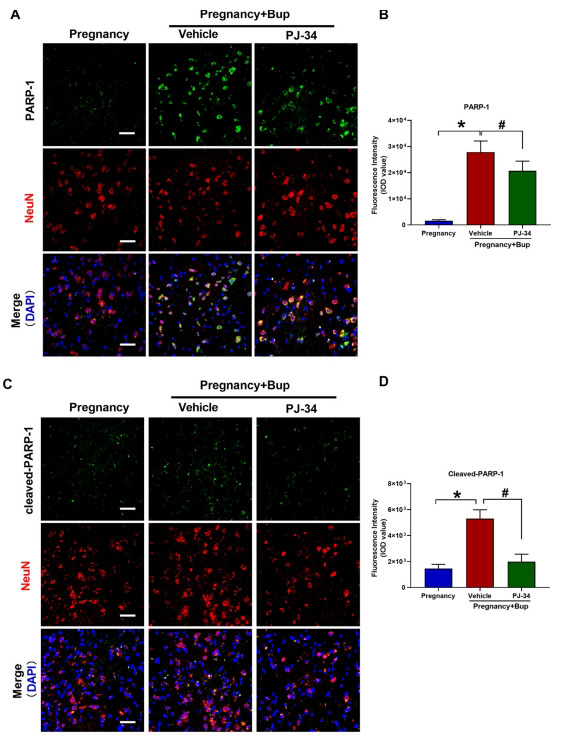
PARP-1 was significantly activated after treatment with bupivacaine in pregnant mice. To verify whether bupivacaine can affect PARP-1 in the spinal cord neurons of pregnant mice, we used immunofluorescence to examine the expression and activation of PARP-1. PJ34, a PARP-1 inhibitor, was administered with bupivacaine in pregnant mice. (**A**, **B**, *p < 0.0001, ^#^*p* = 0.0048) Immunofluorescence was used to examine PARP-1 expression in spinal cord tissue. (**C**, **D**, *p < 0.0001, ^#^*p* < 0.0001) Immunofluorescence was used to measure cleaved- PARP-1 expression in spinal cord tissue. NeuN (red) was used to label mature neurons, and the cell nuclei were stained with DAPI (blue). Scale bar, 50 µm. The results are presented as the mean ± SD (n = 6); *p compared with the pregnancy group; ^#^*p* compared with the PJ-34 group.

In response to oxidative DNA damage, numerous DNA repair enzymes and multiple repair pathways are required to maintain genomic integrity [22]. Proteomics and other studies have shown that nucleic acid excision repair (NER) plays a key role in bupivacaine-medicated neuronal DNA repair [23]. PARP-1 is a multidomain and multifunctional nuclear enzyme regulating chromatin structure and transcription. PARP-1 is a NER enzyme that is activated by oxidative DNA damage. To verify whether bupivacaine could affect PARP-1 in the spinal cord neurons of pregnant mice, we used Western blotting and immunofluorescence to examine the expression and activation of PARP-1. Moreover, the NeuN antibody was used to label spinal cord neurons. As shown in Fig. (**3**), bupivacaine directly increased PARP-1 expression (Figs. **3A** and **B**; fold change 17.9, *p < 0.0001; Supplementary Figs. **S1A** and **B**; fold change 38.7, *p < 0.0001) and PARP-1 activation (Figs. **3C** and **D**; fold change 3.63, *p < 0.0001; Supplementary Fig. **S1A** and **C**; fold change 4.12, *p < 0.0001) in the spinal cord neurons of pregnant mice. The PARP inhibitor PJ34 blocked bupivacaine-induced PARP-1 activation Figs. (**3C** and **3D**; fold change 0.37, ^#^*p* < 0.0001; Supplementary (Figs. **S1A** and **C**); fold change 0.54, ^#^*p* < 0.0001).

**Fig. (4):**
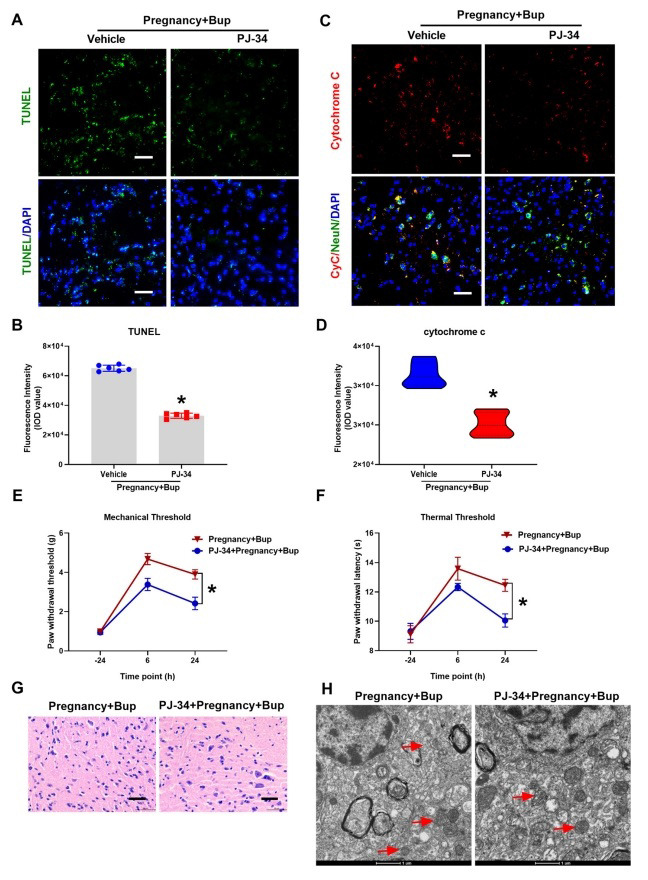
PARP-1 inhibitors could significantly reduce the neurotoxicity induced by bupivacaine in pregnant mice. PJ34, a PARP-1 inhibitor, was administered with bupivacaine in pregnant mice. (**A**, **B**, *p = 0.0344) TUNEL staining was performed to determine cell apoptosis and DNA damage in spinal cord tissues. The cell nuclei were stained with DAPI (blue). Scale bar, 50 µm. (**C**, **D**, *p = 0.0064) Immunostaining of cytochrome c. Spinal cord tissue was probed with anti-cytochrome c (red fluorescence). NeuN (Green) was used to label mature neurons, and the cell nuclei were stained with DAPI (blue). Scale bar, 50 µm. At twenty-four hours before and six and twenty-four hours after bupivacaine injection, behavioral tests for mechanical (**E**, *p < 0.0001) and thermal thresholds (**F**, *p < 0.0001) were performed. (**G**) H/E staining of the spinal cord. Spinal cords were stained with H/E and imaged with light microscopy. Scale bar: 50 µm. (**H**) Representative TEM images of the spinal cords of pregnant mice treated with or without bupivacaine. The red arrows indicate mitochondria. Scale bar: 1 µm. The results are presented as the mean ± SD (n = 6); *p compared with the Preg + Bup group.

Cellular DNA damage can activate PARP-1 and induce PARP-1-dependent cell death [24]. To determine whether bupivacaine-induced spinal neuron injury was triggered *via* the overactivation of PARP-1, we used the PARP-1 inhibitor PJ34. TUNEL assays revealed that PJ34 reduced bupivacaine-induced apoptosis and DNA damage in the spinal cord of pregnant mice (Figs. **4A** and **B**; fold change 0.5, *p = 0.0344). As shown in Figs. (**4C** and **D**) (fold change 0.8, *p = 0.0064), the immunofluorescence results showed that the expression of cytochrome c was decreased after pretreatment with PJ34. Consistent with this finding, the behavioral test results showed that the PWMT (Fig. **4E**, *p < 0.0001) and PWTL (Fig. **4F**, *p < 0.0001) were significantly decreased after pretreatment with PJ34 compared to those in the Preg + Bup group. The results of the H/E staining (Fig. **4G**) showed that pretreatment with PJ34 improved the bupivacaine-induced spinal cord histopathological changes. TEM was used to observe ultrastructural changes in mitochondria. As shown in Fig. (**4H**), less mitochondrial fragmentation with structural fragmentation was observed in the PJ34 group than in the other groups. These findings indicated that inhibiting PARP-1 decreased bupivacaine-mediated DNA damage and spinal neuronal injury in pregnant mice.

### 3.4 Autophagic Flux in Pregnant Mice was Disrupted by Bupivacaine, and an Autophagy Inhibitor Could Reverse this Disruption

**Fig. (5):**
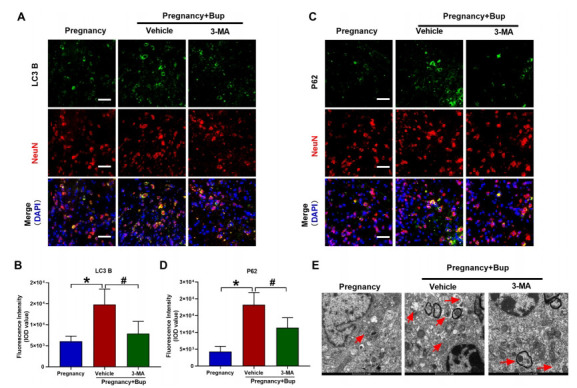
Autophagic flux was disrupted after treatment with bupivacaine in pregnant mice. LC3B and P62 are autophagy markers. 3-MA, an inhibitor of autophagic flux, was administered with bupivacaine in pregnant mice. Immunofluorescence analysis of LC3 B and P62 reflected the dynamic changes in autophagic flux in the spinal cords of pregnant mice. (**A**, **B**, *p = 0.0002, ^#^*p* = 0.0017) Immunostaining of LC3 B. The spinal cord was probed with LC3 B (green fluorescence). (**C**, **D**, *p < 0.0001, ^#^*p* = 0.0025) Immunostaining of P62. The spinal cord was probed with P62 (green fluorescence). NeuN (red) was used to label mature neurons, and the cell nuclei were stained with DAPI (blue). Scale bar, 50 µm. (**E**) The accumulation of enlarged electron-light double-membraned structures in the spinal cords of mice. We performed TEM to evaluate autophagosomes (red arrows). Scale bar: 1 µm. The results are presented as the mean ± SD (n = 6); *p compared with the pregnancy group; ^#^*p* compared with the Preg + Bup group.

It has been previously reported that bupivacaine can trigger autophagy [25]. Our preliminary proteomics results showed that bupivacaine could activate the neuronal autophagy-related mammalian target of rapamycin, the mTOR signaling pathway [26]. However, the role of autophagy in bupivacaine-induced spinal neuronal injury in pregnant mice is unclear. To our knowledge, LC3-B and P62 are autophagy markers. Immunofluorescence analysis of LC3B and P62 indicated dynamic changes in autophagic flux in the spinal cords of pregnant mice. The NeuN antibody was used to label spinal cord neurons. As shown in Figs. (**5A** and **B**) (fold change 2.45, *p = 0.0002), LC3B expression was significantly higher in the spinal cord neurons of the Preg + Bup group. Moreover, the expression of P62 was also increased in the spinal cord neurons of the Preg + Bup group (Figs. **5C** and **D**; fold change 4.24, *p < 0.0001). The autophagic microstructure of the spinal cord was observed by TEM [27]. The TEM results showed that after bupivacaine treatment, many autophagosomes appeared in the spinal neurons of pregnant mice (Fig. **5E**). To further investigate the role of autophagic flux in our model, 3-MA, an inhibitor of autophagic flux, was administered with bupivacaine in pregnant mice. Treatment with 3-MA significantly reduced bupivacaine-induced LC3B (Fig. (**5B**), fold change 0.53, ^#^*p* = 0.0017) and P62 (Fig. (**5D**), fold change 0.63, ^#^*p* = 0.0025) expression. When the animals were pretreated with 3-MA, the number of autophagosomes was also reduced in the Preg+Bup group (Fig. **5E**).

**Fig. (6):**
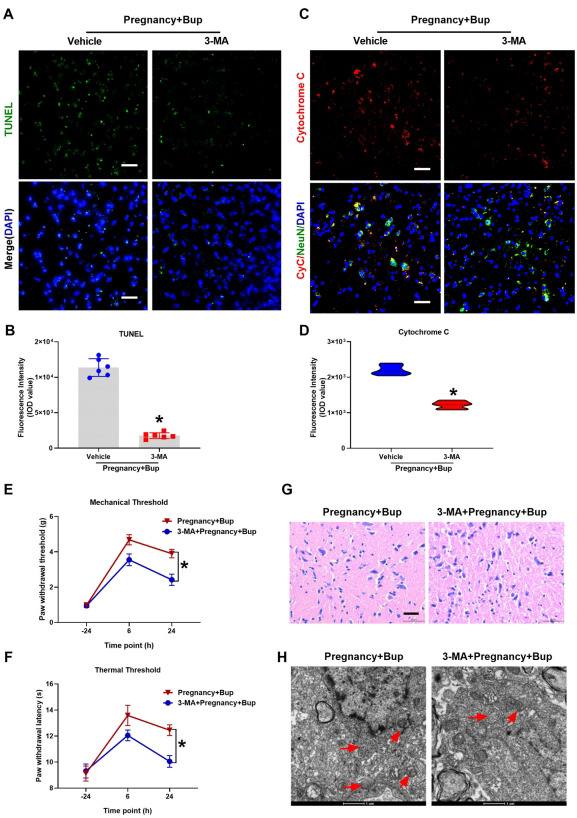
3-MA could significantly reduce the neurotoxicity mediated by bupivacaine. 3-MA was administered with bupivacaine in pregnant mice. (**A**, **B**, *p < 0.0001) TUNEL staining was performed to determine apoptosis and DNA damage in the spinal cord tissue. The cell nuclei were stained with DAPI (blue). Scale bar, 50 µm. (**C**, **D**, *p < 0.0003) Immunostaining of cytochrome c. Spinal cord was probed with anti-cytochrome c (red fluorescence). NeuN (Green) was used to label mature neurons, and the cell nuclei were stained with DAPI (blue). Scale bar, 50 µm. At twenty-four hours before and six and twenty-four hours after bupivacaine injection, the behavioral tests for mechanical (**E**, *p < 0.0001) and thermal thresholds (**F**, *p < 0.0001) were performed. (**G**) H/E staining of the spinal cord. Spinal cords were stained with H/E and imaged under light microscopy. Scale bar: 50 µm. (**H**) Representative TEM images of the spinal cords of pregnant mice treated with or without bupivacaine. The red arrows indicate mitochondria. Scale bar: 1 µm. The results are presented as the mean ± SD (n = 6); *p compared with the Preg + Bup group.

To further confirm the role of autophagic flux in bupivacaine-induced spinal neuronal injury in pregnant mice, 3-MA was administered with bupivacaine to pregnant mice. The TUNEL assay results revealed that 3-MA reduced bupivacaine-induced apoptosis and DNA damage in the spinal cords of pregnant mice (Figs. **6A** and **B**; fold change 0.16, *p < 0.0001) as shown in Figs. (**6C** and **D**) (fold change 0.55, *p < 0.0003), the immunofluorescence results showed that the expression of cytochrome c was decreased after pretreatment with 3-MA. The behavioral test results showed that the PWMT (Fig. (**6E**), *p < 0.0001) and PWTL (Fig. (**6F**), *p < 0.0001) were significantly decreased after pretreatment with 3-MA compared to those in the Preg+Bup group. The results of the H/E staining (Fig. **6G**) showed that pretreatment with 3-MA improved the bupivacaine-induced spinal cord histopathological changes. As shown in Fig. (**6H**), reduced mitochondrial fragmentation with structural fragmentation was observed in the 3-MA group. Overall, these findings indicated that inhibiting autophagic flux decreased bupivacaine-mediated DNA damage and spinal neuronal injury in pregnant mice. In addition, inhibiting autophagic activation may serve as a protective mechanism against bupivacaine-induced cytotoxicity.

### 3.5 CKO of PARP-1 Reduced Neurotoxicity and Alleviated the Disruption of Autophagic Flux Mediated by Bupivacaine in Pregnant *Parp-1*^-/-^ Mice

**Fig. (7):**
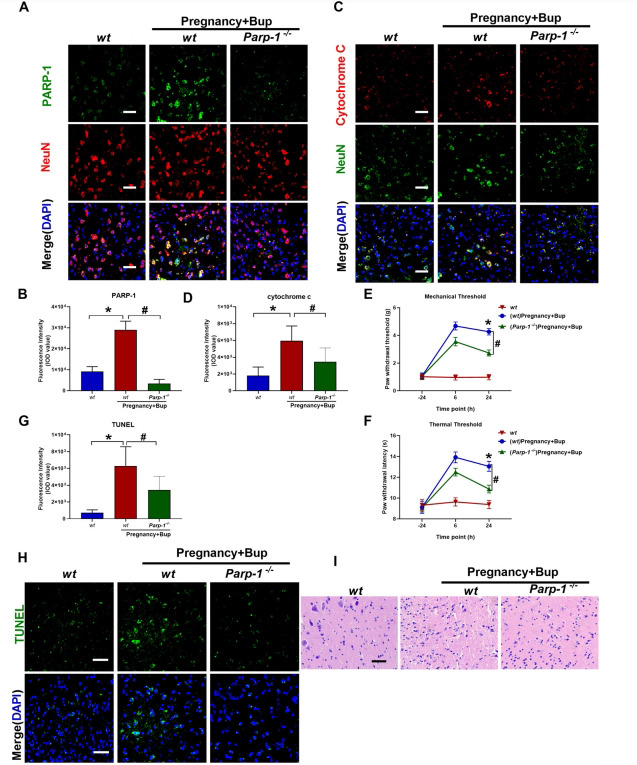
Conditional knockdown of PARP-1 reduced the neurotoxicity mediated by bupivacaine in pregnant mice (*Parp-1*^-/-^). (**A**, **B**, *p < 0.0001, ^#^*p* < 0.0001) Immunofluorescence was used to examine PARP-1 expression in the spinal cord tissues of pregnant *Parp-1*^-/-^ CKO mice (green fluorescence). NeuN (Green) was used to label mature neurons, and the cell nuclei were stained with DAPI (blue). Scale bar, 50 µm. (**C**, **D**, *p = 0.0006, ^#^*p* = 0.0285) Immunostaining of cytochrome c. Spinal cords of pregnant *Parp-1*^-/-^ CKO mice were probed with anti-cytochrome c (red fluorescence). NeuN (Green) was used to label mature neurons, and the cell nuclei were stained with DAPI (blue). Scale bar, 50 µm. At twenty-four hours before and six and twenty-four hours after bupivacaine injection, the behavioral tests for mechanical (**E**, *p < 0.0001, ^#^*p* < 0.0001) and thermal thresholds (**F**, *p < 0.0001, ^#^*p* < 0.0001) were performed. (**G**, **H**, *p = 0.0006, ^#^*p* = 0.0233) TUNEL staining was performed to determine apoptosis and DNA damage in spinal cord tissue of pregnant *Parp-1*^-/-^ CKO mice. The cell nuclei were stained with DAPI (blue). Scale bar, 50 µm. (**I**) H/E staining of the spinal cord. The spinal cords of mice were stained with H/E and imaged under light microscopy. Scale bar: 50 µm. The results are presented as the mean ± SEM (n = 6); *p compared with the WT group; ^#^*p* compared with the (WT) Preg + Bup group.

To further validate the conclusions from the previous analyses, *Parp-1*^flox/flox^ mice were crossed with Nes-Cre transgenic mice to obtain neuronal CKO mice. Consistent with earlier observations, reduced gene expression levels of PARP-1 were observed in pregnant *Parp-1*^-^**^/-^** mice (Figs. **7A** and **B**); fold change 0.12, ^#^*p* < 0.0001; Supplementary Figs. (**S1D** and **E**); fold change 0.08, *p < 0.0001) and reduced PARP-1 activation (Supplementary Figs. **S1D** and **F**; fold change 0.07, ^#^*p* < 0.0001). As shown in Figs. (**7C** and **D**) (fold change 3.3, *p = 0.0006, fold change 0.58, ^#^*p* = 0.0285), the expression of cytochrome c was decreased by bupivacaine treatment in the pregnant *Parp-1*^-^**^/-^** CKO mice compared with the WT mice. The behavioral test results showed that the PWMT (Fig. (**7E**), *p < 0.0001, ^#^*p* < 0.0001) and PWTL (Fig. (**7F**), *p < 0.0001, ^#^*p* < 0.0001) were significantly decreased after bupivacaine treatment in the pregnant *Parp-1*^-^**^/-^** CKO mice. The TUNEL assay results revealed that knockdown of PARP-1 reduced the bupivacaine-induced apoptosis and DNA damage in the spinal cords of pregnant mice (Figs. (**7G** and **H**); fold change 8.8, *p = 0.0006, fold change 0.55, ^#^*p* = 0.0233). The H/E staining (Fig. **7I**) results showed that spinal neuronal knockdown of PARP-1 improved the bupivacaine-induced spinal cord histopathological changes.

**Fig. (8):**
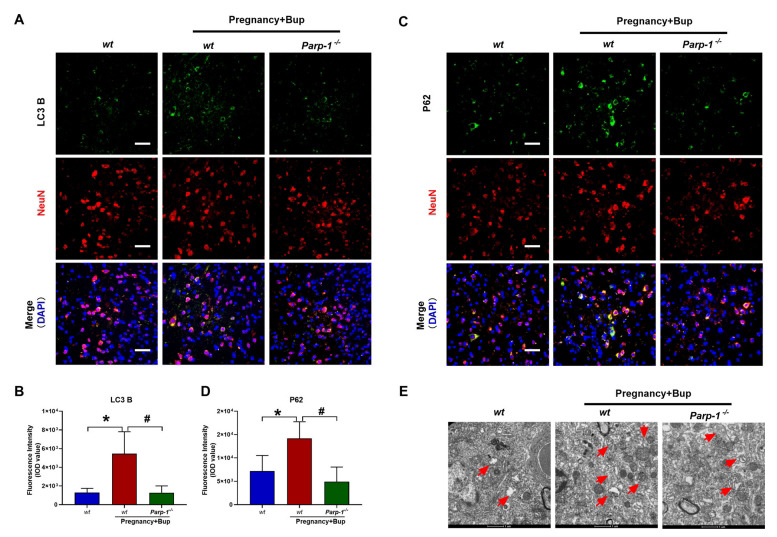
Conditional knockdown of PARP-1 alleviated the disruption of autophagic flux mediated by bupivacaine in pregnant mice (*Parp-1*^-/-^). Immunofluorescence analysis of LC3 B and P62 reflected the dynamic changes of autophagic flux in the spinal cords of pregnant *Parp-1*^-/-^ CKO mice. (**A**, **B**, *p = 0.0005, ^#^*p* = 0.0004) Immunostaining of LC3 B. The spinal cord was probed with LC3 B (green fluorescence). (**C**, **D**, *p = 0.0069, ^#^*p* = 0.0006) Immunostaining of P62. The spinal cord was probed with P62 (green fluorescence). NeuN (red) was used to label mature neurons, and the cell nuclei were stained with DAPI (blue). Scale bar, 50 µm. (**E**) The accumulation of enlarged electron-light double-membraned structures in the spinal cords of pregnant *Parp-1*^-/-^ CKO mice. We performed TEM analysis to evaluate autophagosomes (red arrows). Scale bar: 1 µm. The results are presented as the mean ± SEM (n = 6); *p compared with WT group; ^#^*p* compared with (WT) Preg+ Bup group.

To further evaluate the role of PARP-1 in bupivacaine-induced autophagy, we examined LC3B and P62 expression and observed autophagosomes after bupivacaine treatment in pregnant *Parp-1***^-/-^** CKO mice. As shown in Figs. (**8A** and **B**) (fold change 4.2, *p = 0.0005, fold change 0.23, ^#^*p* = 0.0004), LC3B expression was significantly decreased in the spinal cord neurons of pregnant *Parp-1*^-/-^ CKO mice. Moreover, the expression of P62 was also reduced in pregnant *Parp-1*^-^**^/-^** CKO mice (Figs. (**8C** and **D**); fold change 1.97, *p = 0.0069, fold change 0.35, ^#^*p* = 0.0006). The TEM results showed that after bupivacaine treatment, a small number of autophagosomes appeared in the spinal neurons of pregnant *Parp-1***^-/-^** CKO mice compared with WT mice (Fig. **8E**).

## 4 DISCUSSION

Spinal anesthesia still has several complications associated with obstetric applications, including sensory dysfunction and TNS [28]. Some studies suggested that TNS was simply pain, while others considered TNS as pain and abnormal sensation (hypoesthesia or dysesthesia) [28]. TNS often causes severe pain within 24 hours after spinal anesthesia, negatively impacting patients’ mental and physical health [29]. Sufficient evidence has confirmed that bupivacaine, used for spinal anesthesia, can induce neurotoxic damage in cell and animal models [30]. Our previous study indicated that bupivacaine could mediate neuronal oxidative stress, DNA damage, and apoptosis [20]. We confirmed neurotoxic damage in a pregnant mouse model 24 hours after a bupivacaine injection. During pregnancy, the maternal oxidative imbalance is unquestionably linked to abnormalities in the expectant mother and fetal development, such as TNS, diabetes, or preeclampsia. Late pregnancy may induce transcriptional activation of the peripheral innate immune system and increase oxidative DNA damage among healthy third-trimester pregnant women [31]. We, therefore, focused mainly on whether high maternal oxidative stress during pregnancy is the key to exacerbating the neurologic complications of bupivacaine.

Oxidative stress in neurons may trigger a cascade of events, including activation of the neuronal death receptor signaling pathway, mitochondrial dysfunction, and the loss of antiapoptotic effects, which ultimately cause neuronal apoptosis and oxidative DNA damage [32]. In response to oxidative DNA damage, numerous DNA repair enzymes and multiple repair pathways are required to maintain genomic integrity [22]. Proteomics and other studies have shown that NER plays a key role in bupivacaine-mediated neuronal DNA repair [23]. PARP-1 is a multidomain and multifunctional nuclear enzyme regulating chromatin structure and transcription. Previous studies have shown that oxidative DNA damage stimulates PARP-1 activity. Following DNA damage, PARP-1, a DNA damage sensor, and signaling molecule, quickly relocates to DNA damage sites and catalyzes the transfer of ADP-ribose polymers, facilitating DNA repair [33]. The roles of PARP-1 in single-strand break repair (SSBR), base excision repair (BER), double-strand break (DSB) repair, and the removal of bulky adducts are widely accepted [34]. When cells are subjected to low levels of stress, PARP-1 triggers DNA repair and promotes cell survival, but when under high levels of stress, PARP-1 exacerbates cell death. Some studies have shown that extreme DNA damage results in PARP-1 hyperactivation, ATP depletion, and cell death from bioenergetic collapse [35]. In addition, the same DNA damage can initiate repair and nonapoptotic cell death that depends on PARP-1. These findings suggest that PARP-1 has dual roles. Here, we used a mouse model of pregnancy and investigated whether PARP-1 played a role in oxidative stress-induced DNA damage. As revealed by histochemical and immunohistochemical examinations, bupivacaine activated ROS in spinal tissue sections, which led to DNA damage, PARP-1 activation, and apoptosis. In this study, PARP-1 CKO in the neurons of pregnant mice showed a reduction in nerve damage after bupivacaine treatment. These results indicated a positive effect of PARP-1 inhibition or genetic deletion on neurologic outcomes after parturition.

PARP-1 activation is known to induce cell injury, but the mechanisms underlying this induction are poorly understood. Previous studies have shown a close relationship between PARP-1 activation and autophagy [36]. Under oxidative DNA damage conditions, PARP-1 determines the fate of cells by regulating the dynamic balance between autophagy and necrosis. Previous studies have indicated that PARP-1 activation is involved in optimizing autophagy during hunger-induced autophagy by inhibiting the ROS production/DNA damage/NAD^+^ consumption axis.

While autophagy is an essential process for cell homeostasis, excessive autophagy triggers cell death programs and the activation of apoptotic signaling pathways, indicating the dual roles of autophagy in survival [37]. To assess the pathophysiological role of autophagy in neurologic complications after spinal anesthesia, we evaluated the protein levels of LC3B and P62. We used TEM to examine autophagosomes and monitor autophagic flux. However, autophagic flux is a dynamic and complex process that involves the formation of autophagosomes, delivery of autophagic substrate lysosomes, and degradation of these substrates inside lysosomes [38]. A recent study showed that PARP-1 promotes autophagosome formation during the initiation stage but inhibits autolysosome formation, demonstrating that PARP-1 is responsible for impairing late-stage autophagy in particular [11]. In the present study, to understand the roles of PARP-1 in bupivacaine-induced dysregulation of autophagic flux, the autophagy markers LC3B and P62 were evaluated in PARP-1 CKO mice. The results showed that both LC3B and P62 expression were decreased in PARP-1 knockout mice, which indicated that PARP-1 might affect autophagy either by stimulating autophagosome formation or controlling the degradative function of lysosomes. However, the intracellular mechanisms by which PARP-1 activation fine-tunes autophagic flux initiation remain poorly understood.

PARylation is a posttranslational modification (PTM) that may also regulate additional downstream autophagy events [39]. PARP-1 is the main acceptor protein for poly (ADP-ribosyl) action *in vivo*, mediating PARylation activity [40]. A previous study reported that PARP-1-catalyzed PARylation induces the dissociation of the PARP-1/AMPK complex to activate autophagy. Singh *et al.* showed that PARP-1 activity is facilitated through the cytosolic translocation of HMGB1 *via* PARylation, which is known to induce autophagy [41]. Thus, our further research goal is to show that PARP-1-mediated PARylation of autophagy proteins regulates spinal cord neuronal injury in pregnancy.

The overactivation of PARP-1 and simultaneous disruption of autophagic flux eventually led to neuronal injury. Additional results revealed that PARP-1 knockdown and autophagy inhibition could alleviate bupivacaine-mediated neurotoxicity in pregnant mice. These findings may be a reference for treating bupivacaine-mediated neurotoxicity in pregnancy, especially in TNS.

## CONCLUSION

In conclusion, we first observed that the local anesthetic bupivacaine aggravated the neurotoxic injury in pregnancy. A novel molecular pathway was proposed in which oxidative stress-mediated DNA damage activated PARP-1, which induced autophagic flux blockade. Ultimately, these events led to neuronal damage (Graphical abstract). From the present results, the regulation of spinal cord neuronal injury in pregnancy by PARP-1 inhibition emerges as a promising therapeutic target that remains understudied. The mechanisms of this mode of regulation will lay the groundwork for subsequent studies on bupivacaine-induced TNS in pregnancy.

Our *in vivo* studies are limited to the neurotoxic effects of local anesthetics, and we did not take into account the effects of sex hormones that may or may not impact neurotoxic injury in pregnancy. This is a limitation of the present study. Hormonal changes intrinsic to pregnancy may alter pain sensitivity. Existing literature points to when estrogen levels are constantly elevated, as in pregnancy, pain sensitivity is known to decrease [42]. In addition, many established studies have shown that pregnancy-specific hormones such as estrogen and progesterone play a role in postsynaptic maintenance and neuroprotective properties [43]. We can thus speculate that hormonal fluctuations during pregnancy may play a protective role rather than aggravate neurotoxic injury.

We were especially interested in PARP inhibitors, which have been shown to protect against injury in various animal models, possibly through increased NAD^+^ levels [44]. PARP-1 inhibitors (3-aminobenzamide and 4-hydroxyquinazoline) prevent hydrogen peroxide (H_2_O_2_)-induced ATP depletion with a reversion of the mode of cell death from necrosis back to apoptosis [45]. PARP-1 inhibitors play an important role in numerous inflammation-related diseases by inhibiting inflammatory responses *via* inhibition of NF kappa B activation. COVID-19 is spreading widely around the world. Post-COVID-19 inflammation is a very complex network system that has a decisive influence on the prognosis of patients [46]. Currently, no promising treatment options are available against COVID-19 that can be recommended globally. Published studies have shown that PARP-1 inhibition limits inflammation-induced tissue damage, including acute lung injury in animal models, and PARP-1 inhibitors have therefore been discussed as a potential treatment option for COVID-19 [47].

PARP-1 enhances autophagy through the AMPK–mTOR pathway, and its activity and knockdown can promote and prevent stress-mediated autophagy, respectively [48]. In a broad sense, there are four different forms of autophagy: macroautophagy, microautophagy, chaperone-mediated autophagy (CMA), and noncanonical autophagy [49]. However, since these detailed variations are not discussed here, we simply defined “autophagic degradation activity” using autophagic flux [50]. Autophagy was quantified by measuring LC3B/P62, a classical marker of autophagic flux. Notably, LC3B is not a specific autophagy marker because it can be linked to nonautophagosome membranes [51, 52]. This study indicates that PARP-1 mediates the autophagy pathway, but whether PARP-1 directly or indirectly mediates the autophagy pathway needs to be further studied.

## Data Availability

Not applicable.

## LIST OF ABBREVIATIONS

IOD: Integral Optic Density
PWT: Paw Withdrawal Threshold
ROS: Reactive Oxygen Species
SOD: Superoxide Dismutase
TEM: Transmission Electron Microscopy
TNS: Transient Neurological Symptoms
