# Supplementary material for: Oxidative DNA Damage-induced PARP-1-mediated Autophagic Flux Disruption Contributes to Bupivacaine-induced Neurotoxicity During Pregnancy
Source: Curr Neuropharmacol. 2023 Aug 15;21(10):2134–50. doi: 10.2174/1570159X21666230404102122 (PMC10556365; doi:10.2174/1570159X21666230404102122)
Supplement: Supplementary file 1 [file CN-21-2134_SD1.pdf]

Supplementary Material

Oxidative DNA Damage-induced PARP-1-mediated Autophagic Flux Disruption Contributes to Bupivacaine-induced Neurotoxicity During Pregnancy

Jiaming Luo[1,#], Lei Zeng[2,#], Ji Li[1], Shiyuan Xu[1,*] and Wei Zhao[1,*]

[1]Department of Anesthesiology, Zhujiang Hospital, Southern Medical University, Guangzhou City, Guangdong Province, China; [2]Division of Laboratory Science, Affiliated Cancer Hospital & Institute of Guangzhou Medical University, Guangzhou, China

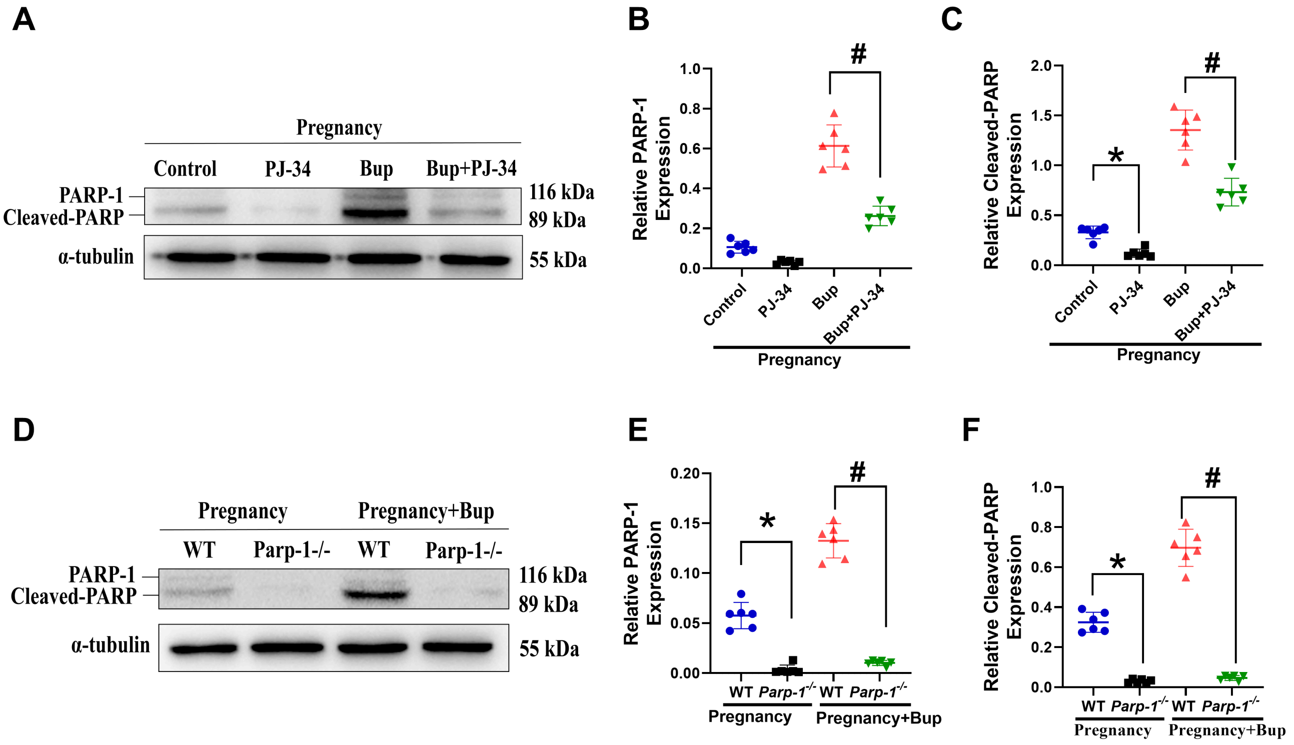

Supplemental Fig. (S1). Inhibition and conditional knockdown of PARP-1 downregulated the expression of PARP-1 caused by bupivacaine in pregnant mice. To verify whether inhibition can affect PARP-1 expression and activation in the spinal cord of pregnant mice after exposure to bupivacaine, western bloting was used to examine the PARP-1 and ecleaved-PARP-1 expression in the spinal cord tissues of pregnant mice. PJ34, a PARP-1 inhibitor, was administered with bupivacaine in pregnant mice (A, B, C; $*p < 0.0001$, $^{\#}p < 0.0001$). Western bloting was used to assay PARP-1 and ecleaved-PARP-1 expression in the spinal cord tissues of pregnant *Parp-1*$^{-/-}$ CKO mice (D, E, F; $*p < 0.0001$, $^{\#}p < 0.0001$). The blots were quantified by a densitometric method. The results are presented as the mean ± SEM (n = 6); $*p$ compared with Control/WT group; $^{\#}p$ compared with (WT) Preg + Bup group.

1875-6190/23 $65.00+.00 © 2023 Bentham Science Publishers
